# Robust Pedestrian Detection and Intrusion Judgment in Coal Yard Hazard Areas via 3D LiDAR-Based Deep Learning

**DOI:** 10.3390/s25185908

**Published:** 2025-09-21

**Authors:** Anxin Zhao, Yekai Zhao, Qiuhong Zheng

**Affiliations:** College of Communication and Information Technology, Xi’an University of Science and Technology, Xi’an 710054, China; zhaoanxin@126.com (A.Z.); 23307223001@stu.xust.edu.cn (Q.Z.)

**Keywords:** LiDAR, deep learning, 3D object detection, regional intrusion, hierarchical judgment, coal yard

## Abstract

Pedestrian intrusion in coal yard work areas is a major cause of accidents, posing challenges for the safe supervision of coal yards. Existing visual detection methods suffer under poor lighting and a lack of 3D data. To overcome these limitations, this study introduces a robust pedestrian intrusion detection method based on 3D LiDAR. Our approach consists of three main components. First, we propose a novel pedestrian detection network called EFT-RCNN. Based on Voxel-RCNN, this network introduces an EnhancedVFE module to improve spatial feature extraction, employs FocalConv to reconstruct the 3D backbone network for enhanced foreground–background distinction, and utilizes TeBEVPooling to optimize bird’s eye view (BEV) generation. Second, a precise 3D hazardous area is defined by combining a polygonal base surface, determined through on-site exploration, with height constraints. Finally, a point–region hierarchical judgment method is designed to calculate the spatial relationship between pedestrians and the hazardous area for graded warning. When evaluated on the public KITTI dataset, the EFT-RCNN network improved the average precision for pedestrian detection by 4.39% in 3D and 4.68% in BEV compared with the baseline, while maintaining a real-time processing speed of 28.56 FPS. In practical tests, the pedestrian detection accuracy reached 92.9%, with an average error in distance measurement of 0.054 m. The experimental results demonstrate that the proposed method effectively mitigates complex environmental interference, enables robust detection, and provides a reliable means for the proactive prevention of pedestrian intrusion accidents.

## 1. Introduction

As a critical node in coal energy infrastructure, coal yards require advanced sensing solutions to mitigate the constant safety hazards associated with complex spaces and low visibility. Traditional surveillance systems rely on cameras, but they have limitations in terms of pedestrian detection in dark and occluded areas. While thermal cameras have advantages in low-light conditions, they lack the accurate 3D spatial data provided by LiDAR and their performance may be degraded by environmental factors such as dust, which is prevalent in coal yards. Coal yard work areas, especially under conveyor bridges, face a constant threat from coal pile landslides and mechanical equipment. The existing methods only play roles in summarizing safety-related accidents afterwards, and cannot actively prevent pedestrian intrusion accidents [1,2].

Detecting intrusions into hazardous areas is a top priority for enhancing safety. Various industries have implemented numerous measures to detect workers who intrude into hazardous areas. Among these safety measures, traditional management methods such as safety education and safety inspections are predominant. These rely mainly on workers’ self-awareness and supervisors’ inspections, which are both time-consuming and labor-intensive, and also require full-process management [3]. With the development of machine vision technology, the replacement of manual inspection with machines has been increasingly applied. At present, the two mainstream approaches for regional intrusion detection are detection based on vision cameras [4,5] and detection based on LiDAR [6,7,8,9]. Of these, detection based on visual cameras mainly relies on passive visual cameras to collect front images. The cameras can capture the color information of objects, with fast collection speed and rich data acquisition; however, these methods have several limitations. First, the data obtained by cameras are greatly affected by illumination. For example, additional light needs to be supplemented at night, and the visual range is reduced in rainy and foggy weather. In addition, the image data do not contain spatial information, making it difficult to determine the specific spatial relationship between pedestrians and hazardous areas [10,11]. During the data acquisition process, LiDAR is less affected by illumination, provides depth information, and has the advantages of a wide detection range and high distance resolution. Furthermore, the deployment of lidar systems usually requires point cloud compression algorithms for processing to facilitate storage and transmission [12,13]. Despite these challenges, LiDAR is still widely used in environmental detection systems for autonomous driving, ports, and railways [14,15,16,17], owing to its robustness in complex environments. Considering that coal yard environments are relatively enclosed and have poor lighting conditions at night, vision-based detection methods are prone to false and missed detections. Therefore, a LiDAR-based detection method was selected in this research. To the best of our knowledge, this is the first time that LiDAR has been applied to achieve regional intrusion detection in coal yards.

Detection of intrusion in a region involves two main steps: object detection and intrusion judgment. Object detection is the initial stage in region intrusion detection. Algorithms that extract foreground objects through traditional point cloud gridding [18] and clustering [19,20] have been widely used in practical object detection. Since the coal yard ground contains water and the background is complex, traditional detection algorithms lack robustness and are easily interfered with by objects such as accumulated ground water and transport platforms, leading to false and missed detections. As autonomous driving technology advances, 3D point cloud deep learning-based object detection algorithms have gained prominence. These deep learning algorithms offer enhanced robustness and high recognition accuracy. They can be categorized into one-stage and two-stage methods, based on their detection paradigm. Single-stage detectors achieve efficient inference through an end-to-end architecture. VoxelNet [21] realizes geometric feature extraction through regular voxel encoding, but its 3D convolution has a high computational cost. SECOND [22] includes a sparse convolution operation to effectively reduce the computational complexity of voxel features. PointPillars [23] uses pillar coding to eliminate redundant calculations along the *z*-axis and significantly improve computational efficiency. PillarNet [24] further optimizes the pillar representation paradigm and proposes cross-layer feature fusion, enhancing the object detection ability in complex scenarios while maintaining real-time performance. VoxelNext [25] utilizes a fully sparse network architecture to break through the performance bottleneck of traditional dense detection heads. Although these methods have the advantage of real-time performance, there is still room for improvement in the positioning accuracy in complex scenarios. Two-stage detectors achieve accuracy breakthroughs through the cascaded structure of region proposal and refined regression. PV-RCNN [26] fuses point clouds and voxels, adds features of key points to corresponding voxels, and performs two-stage fine regression. Part-A^2^ [27] includes a two-stream attention aggregation module that optimizes the feature representation of proposal boxes through foreground-aware and spatial context enhancement strategies. PointRCNN [28] directly generates proposal boxes on the original point clouds, but is limited by the unstructured characteristics of point clouds, resulting in a sharp increase in computational complexity.

In contrast, Voxel-RCNN [29] introduces algorithmic advancements via sparse voxel encoding and feature aggregation. A lightweight backbone network initially generates high-quality proposal boxes, followed by hierarchical feature fusion to refine these boxes. This method innovatively balances the spatial sparsity of 3D point clouds and the effectiveness of feature representation. Considering both accuracy and speed, this study selects Voxel-RCNN as the baseline network and, on this basis, innovatively proposes the pedestrian object detection network EFT-RCNN.

After achieving object detection, the next problem to be solved is completing intrusion judgment within a given hazardous area. The concept of intrusion judgment is not new [30,31,32]. Miao et al. [33] constructed an airborne LiDAR point cloud processing framework, ALORID, and judged intruding objects by calculating the velocity and contour similarity of dynamic objects using the LiDAR time-domain cumulative map. Darwesh et al. [34] detected vehicle positions based on LiDAR point clouds, tracked objects using DBSCAN clustering and the Hungarian algorithm, and judged whether the objects intruded into the hazardous area of the work zone through position and trajectory analysis. Shi et al. [35] segmented moving objects using the background subtraction method based on LiDAR point cloud data, and combined radius filtering pre-processing and deep learning recognition to achieve a real-time alarm for foreign object intrusion. Wu et al. [36] used the Kalman filter to predict vehicle trajectories and combined a two-layer judgment mechanism of a preset warning zone and a detection zone: an intrusion alarm is triggered when the distance of a vehicle entering the warning zone is less than or equal to the threshold. Heng et al. [37] used a real-time locating system (RTLS) to track the coordinate positions of pedestrians, compared these coordinates with a virtual hazardous area model, automatically triggered intrusion warnings, and recorded response behaviors. Ma et al. [38] used LiDAR to detect the coordinates of intruding objects, linked a PTZ camera to capture the target area, identified the types of wild animals using the YOLO algorithm, and triggered alarms.

Analysis of the existing LiDAR-based intrusion judgment methods reveals that their intrusion judgment mechanisms have inherent limitations: alarms are only triggered when an intrusion actually occurs, resulting in a significant delay in security response and failing to provide sufficient time for staff to react; therefore, predicting potential intrusion threats is more significant than developing real-time alarm systems. In this study, by utilizing the spatial position information of 3D point clouds and analyzing the distance relationship between the hazardous area and the object pedestrians, an innovative point–region hierarchical judgment method with a prejudge layer, a warning layer, and an alarm layer is constructed. This method aims to predict pedestrian intrusion threats more accurately and over a longer period, thus preventing accidents.

In summary, to address the core problem of frequent accidents caused by pedestrian intrusion into hazardous areas in high-risk operation environments of coal yards, this study proposes a robust pedestrian detection and hierarchical early-warning method based on LiDAR. The core innovations are as follows:

- **A 3D point cloud object detection network, EFT-RCNN, is proposed.** This network takes Voxel-RCNN as the baseline and makes three key improvements to address the interference of widespread complex backgrounds in coal yard environments on pedestrian detection. (a) An EnhancedVFE module is proposed to enhance the ability to extract geometric features of voxel data. (b) The FocalConv is employed to reconstruct the 3D backbone network, focusing on the feature learning of the foreground regions and suppressing the noise from the cluttered background. (c) TeBEVPooling is applied to optimize the generation of a bird’s eye view and improve the quality of feature fusion.
- **A point–region hierarchical judgment method is proposed.** This method analyzes the spatial relationship between pedestrians and the hazardous area in a progressive manner through the prejudge layer, warning layer, and alarm layer, avoiding the limitations of traditional single-step intrusion judgment and more effectively preventing accidents.

The remainder of this article is organized as follows: The proposed EFT-RCNN network architecture is elaborated in Section 2, including the design principles of the EnhancedVFE module, the backbone network based on FocalConv, and the TeBEVPooling module. The design principle of the point–region hierarchical judgment method is also explained. Section 3 presents the experiments and results. First, comprehensive comparative experiments and ablation studies are conducted on the public dataset KITTI to quantitatively evaluate the accuracy and efficiency of EFT-RCNN in pedestrian detection. Subsequently, the system is deployed in the actual coal yard environment, and comparative experiments on pedestrian intrusion detection under multiple scenarios are conducted. Additionally, the accuracy of the point–region hierarchical judgment method in measuring the intrusion distance is quantitatively evaluated using static pedestrian detection tests. In Section 4, the study is summarized and future research directions are proposed.

## 2. Methods

### 2.1. Offline Training and Online Detection

**Figure 1:**
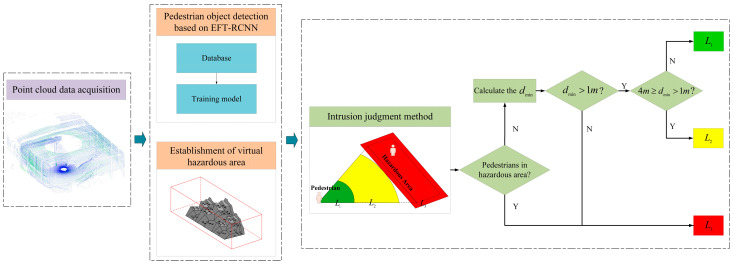
Technical research route. First, acquire the point cloud data for the coal yard. Then, use the pre-trained pedestrian object detection model EFT-RCNN to detect pedestrians and simultaneously establish a virtual hazardous area. Finally, use the hierarchical judgment method to obtain the grade and distance of the pedestrians from the hazardous area. Here, $d_{min}$ represents the minimum distance of the pedestrian from the boundary of the hazardous area, $L_{1}$ is the pre-judgment layer, $L_{2}$ is the warning layer, and $L_{3}$ is the alarm layer.

This study adopts the idea of “Offline Training and Online Detection” and proposes a method for detecting pedestrian intrusion in the hazardous area of a coal yard based on LiDAR point clouds, as shown in Figure 1. Before online detection, the pedestrian object detection network EFT-RCNN needs to be trained offline on the dataset to enable pedestrian object detection. Then, it enters the first stage of online detection: point cloud features are extracted and input into the pretrained model EFT-RCNN for pedestrian object identification. Meanwhile, the bottom surface of the hazardous area polygon is determined according to the fixed coordinates of the coal yard environment and, combined with the height constraint, the polygonal prism of the hazardous area is obtained. This is followed by the second stage of online measurement, intrusion judgment: utilizing the orthogonal projection method, this study maps the pedestrian object and the hazardous zone onto a 2D plane within a unified coordinate system. This approach simplifies the analysis of the intrusion issue by focusing on the spatial interaction between points and polygons in the 2D plane. In order to determine the positional relationships between points and polygons, a point–region hierarchical judgment method is proposed. If the point is an inner point, it is assigned to the L3 alarm layer. For outer points, the minimum distance from the point to the polygon is calculated to determine whether it belongs to the L1 prejudge layer or the L2 warning layer. This method takes into account the environmental characteristics of the coal yard and fully utilizes the spatial positional relationship between pedestrians and the hazardous area, which helps to meet the requirements for safety supervision in the hazardous area.

### 2.2. Pedestrian Object Detection Network EFT-RCNN

#### 2.2.1. EFT-RCNN

Voxel-RCNN first calculates the intensity and average of the 3D coordinates of the input voxel grids using Mean Voxel Feature Encoding (MeanVFE) to complete the voxelization operation. It then constructs a 3D backbone network using sparse convolution blocks to extract voxel features. Following this, HeightCompression is applied to compress the 3D voxel characteristics into 2D features along the height axis, finalizing the transformation from sparse tensors to the bird’s eye view (BEV). Next, a 2D backbone network is employed to capture BEV image attributes and produce Region of Interest (RoI) proposals. Ultimately, it employs Voxel RoI Pooling to refine the RoI proposals with voxel characteristics derived from the 3D backbone network, enhance the feature input to the detection head, and achieve the detection outcomes. However, Voxel-RCNN loses a significant amount of key information during the process of voxelization and converting 3D voxel features to the BEV—particularly in the scenario in this study, where the object pedestrian has limited point cloud information. Direct voxelization processing leads to feature loss and reduced detection accuracy.

**Figure 2:**
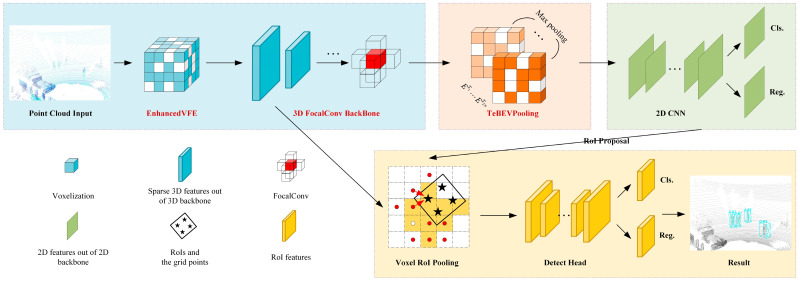
Overall architecture of EFT-RCNN. The blue section pertains to point cloud voxelization and voxel feature extraction. The orange section converts 3D sparse tensors into 2D BEV images. The green section focuses on 2D feature extraction and detection of BEV images. Finally, the yellow section represents the 3D RoI detection head. The text in red indicates the improved module.

As shown in Figure 2, this study proposes a novel object detection network, EFT-RCNN, based on Voxel-RCNN for the high-risk working environments in coal yards: (a) An EnhancedVFE module is proposed in the VFE part to optimize the voxel division of VFE, aggregate local and global features, and improve the quality of feature representation by utilizing feature information at different levels. (b) The 3D backbone network structure is reconstructed by introducing the FocalConv block to dynamically weight the features of each voxel, enhancing the discrimination between the foreground and the background and providing more effective information for pedestrian object detection. (c) In the MAP to BEV part, Height Compression is replaced with Transformation-Equivariant BEV Pooling (TeBEVPooling), which can improve the efficiency and quality of feature fusion, suppress the noise of complex backgrounds, and retain more comprehensive geometric information.

#### 2.2.2. EnhancedVFE

**Figure 3:**
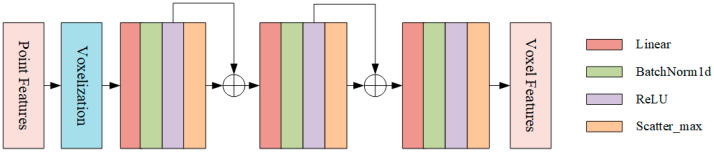
EnhancedVFE module.

VFE is a feature encoding layer that performs the calculation from point cloud features to voxel features. The baseline network adopts the MeanVFE method, which utilizes cubes of a uniform size for voxel partitioning and computes voxel features by averaging all points within each voxel. Although the mean calculation is still valid when the number of points in a single voxel is small, the features may not be stable enough; therefore, the study proposes the EnhancedVFE method (Figure 3). By calculating the offset and concatenating it with other features, it makes up for the deficiency of MeanVFE in feature utilization. It also realizes the aggregation of local and global information and uses feature information at different levels to improve the quality of feature representation. EnhancedVFE can be divided into two steps:

**Voxelization:** For the input point cloud, the point cloud is represented as $P={\left\{p_{i}\right\}}_{i=1}^{N}$, where each point is represented as $p_{i}=(x_{i},y_{i},z_{i},feat_{i})$. It is partitioned into a regular 3D grid of voxels of uniform size. Assuming the given point cloud range is $R=(\min_{x},\min_{y},\min_{z},\max_{x},\max_{y},\max_{z})$, and the depth, height, and width of each voxel are $\left(v_{D},v_{H},v_{W}\right)$, respectively, the number of voxel grids generated by the 3D voxelization result of the entire data on each coordinate is as follows:

(1) $$\left(\frac{\max_{x}-\min_{x}}{v_{D}},\frac{\max_{y}-\min_{y}}{v_{H}},\frac{\max_{z}-\min_{z}}{v_{W}}\right)$$

The point set in a single voxel grid can be represented as follows:

(2) $$V={\left\{x_{i},y_{i},z_{i},feat_{i}\in {\mathbb{R}}^{4}\right\}}_{i=1,2,\cdots ,t},t\geq 0$$

where t represents the number of points in a single voxel grid. First, the study generates a mask based on the number of points in the voxels, which is used to directly set the features of voxel grids without points to 0. Subsequently, the scatter_mean operation is used to calculate the average $\left(v_{x},v_{y},v_{z}\right)$ of all points within the voxel as the centroid of the voxel grid. Finally, the offsets $f_{center}$ and $f_{cluster}$ of each point from the voxel center and the voxel centroid are calculated, and these features are concatenated with the original point features to enhance the feature representation ability:

(3) $$f_{t}=concat(feat_{i},f_{center},f_{cluster})$$

**Voxel feature aggregation:** Feature extraction and aggregation are performed through three PFN layers. For each PFN layer, the features undergo linear transformation, normalization, activation, and scatter_max pooling operations. It is worth noting that, in the first and second PFN layers, the original point features are concatenated with the pooled voxel features. This approach not only preserves local information but also integrates the global information of voxels, enabling more effective extraction and representation of point cloud features and providing higher-quality feature inputs for subsequent 3D perception tasks.

#### 2.2.3. Reconstruction of 3D Backbone by FocalConv

The 3D backbone network is responsible for advanced feature learning in voxel space. Traditional 3D sparse convolutions [22,39] face a trade-off between computational efficiency and receptive field size. To better extract features of foreground pedestrians while suppressing background interference in cluttered environments such as coal yards, we employ FocalConv [40] to reconstruct the Voxel-RCNN backbone.

**Figure 4:**
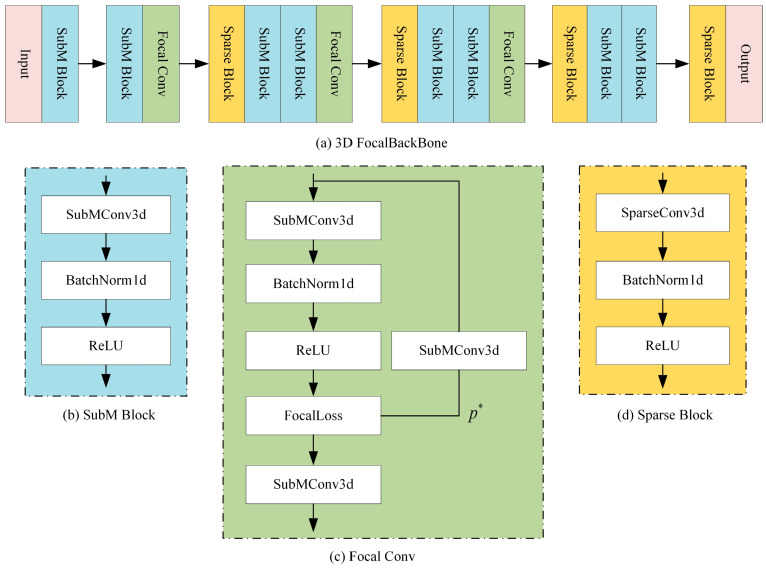
**Three-dimensional** backbone network based on focal convolution. (**a**) The overall structure of the 3D backbone network based on FocalConv. (**b**) The submanifold sparse convolution block. This convolution block ensures sparsity while only adding a relatively small amount of computational burden. (**c**) The focal convolution. This convolution block has two branches. The structure of the main branch is similar to that of the sub-flow sparse convolution block, while its side branch can assist the main branch in distinguishing difficult samples by independently predicting weights. (**d**) A spatial sparse convolution block with a larger receptive field to extract deeper information.

As shown in Figure 4, the 3D backbone network based on focal convolution consists of input and output convolutional layers and four feature extraction layers. It receives voxel features (N, 192) as input. First, a SuBM block is used to compress the 192-dimensional features into 16-dimensional features; subsequently, multi-scale features are extracted through multi-layer Sparse and SuBM blocks, and downsampling operations of 1×, 2×, 4×, and 8× are performed to increase the number of feature channels. Finally, a 128-dimensional feature map is output. In this study, FocalConv is integrated at the end of the first three convolutional blocks, as shown in Figure 4c. It maps the features on the path to 27 dimensions through a unique SubMConv3d and predicts the importance of each voxel. FocalLoss then weights the features on the path according to their predicted importance, enhancing the expression of important features and suppressing the interference of unimportant features. This enables the network to effectively distinguish and extract useful foreground information in complex background environments. Specifically, the main path performs standard sub-convolution operations, the side branch predicts weights $p^{*}$ through an independent convolution, and FocalLoss combines the two to focus on hard-to-classify samples and improve feature discrimination. The Focal 3D backbone network effectively enhances the feature extraction ability for sparse 3D data while maintaining computational efficiency through the combination of 3D sparse convolution and FocalConv. The background of the coal stockyard area is chaotic, which affects the target detection network’s ability to extract foreground pedestrian features. Through this design, target personnel can be clearly distinguished and extracted.

#### 2.2.4. Reconstruction of MAP to BEV by TeBEVPooling

**Figure 5:**
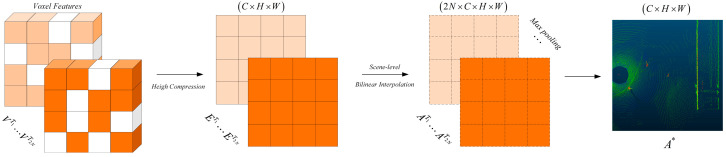
TeBEVPooling method. This process involves transforming voxel features under different rotations or flips, aligning them to a unified coordinate system via bilinear interpolation, and aggregating them through max pooling to generate robust BEV feature maps.

The 3D voxel features are transformed into a 2D feature map using MAP_to_BEV, enabling the application of a well-established 2D convolutional neural network for feature extraction and object detection. The method adopted by the baseline network involves compressing the voxel features in the height dimension and converting the sparse tensor into a dense tensor, ultimately obtaining a 2D feature map. TeBEVPooling is one of the methods proposed by Wu et al. [41]. It aims to align and aggregate multi-channel-transformed voxel features into a compact BEV representation. Its core function is to ensure the consistency between the detection results and the transformation of the input point cloud through the explicit modeling of rotation and reflection transformations, while reducing computational complexity. The study first added the TeBEVPool module to the Voxel RCNN network to implement the MAP_to_BEV operation, as shown in Figure 5. TeBEVPooling is divided into two main steps:

**Feature alignment:** Coordinate alignment is performed on voxel features from different transformation channels (such as features after rotation or flipping), and the BEV features under different transformations are mapped to the same coordinate system through bilinear interpolation. Specifically, the voxel features V are first transformed based on different rotation and flipping angles, which can be formally expressed as ${\left\{V^{T_{j}}\right\}}_{j=1}^{2N}$, as shown in Equation (4). Subsequently, they are compressed along the height dimension to obtain BEV features ${\left\{E^{T_{j}}\right\}}_{j=1}^{2N}$, as shown in Equation (5). Since the BEV features are obtained under different transformations, they need to be aligned to the same coordinate system. A set of scene-level grid points $E^{T_{j}}$ are generated in the coordinate system $X^{T_{j}}$, and according to the transformation operations, the grid points are converted into a new set of grid points ${\left\{X^{T_{j}}\right\}}_{j=1}^{2N}$ in the BEV coordinate system, as shown in Equation (6). Finally, a series of bilinear interpolations I(.,.) are applied on the BEV features to obtain a set of aligned features ${\left\{A^{T_{j}}\right\}}_{j=1}^{2N}$, as shown in Equation (7).

(4) $$V\overset{T_{j}}{\underset{2N}{\to }}{\left\{V^{T_{j}}\right\}}_{j=1}^{2N}$$

(5) $${\left\{V^{T_{j}}\right\}}_{j=1}^{2N}\overset{height}{\to }{\left\{E^{T_{j}}\right\}}_{j=1}^{2N}$$

(6) $${\left\{E^{T_{j}}\right\}}_{j=1}^{2N}\overset{scene-level}{\to }{\left\{X^{T_{j}}\right\}}_{j=1}^{2N}$$

(7) $${\left\{A^{T_{j}}\right\}}_{j=1}^{2N}=I(X^{T_{j}},E^{T_{j}})$$

**BEV feature aggregation:** To improve efficiency, max pooling is applied to the 2N aligned feature maps to extract the most prominent features from the aligned features and generate a lightweight BEV feature map $A^{*}$.

In addition to pedestrian objects, the coal yard environment may also contain other objects, such as coal piles and coal charging cars. In this case, traditional detection methods are prone to miss pedestrian objects. However, TeBEVPooling significantly improves object detection accuracy by performing multichannel transform feature alignment and aggregation, which preserves more comprehensive geometric information. TeBEVPooling aggregates multi-transform features through maximum pooling, effectively highlighting key pedestrian features and suppressing noise from complex backgrounds. In addition, TeBEVPooling exhibits transformation equivariance and is robust to rotation, making it suitable for LiDAR systems installed at an angle, requiring only the application of appropriate coordinate transformations.

### 2.3. Point–Region Hierarchical Judgment Method

The division of hazardous areas is a prerequisite for realizing intrusion judgment. This study proposed a hazardous area division method using a parameterized clipping box, which is simple and effective. The core idea is that the hazardous area is jointly constructed by a polygon on the base-plane (XOY) and height constraints. First, with the installation position of the LiDAR as the coordinate origin, the vertex coordinates of the base-plane polygon in the XOY plane are determined based on on-site survey data to accurately map the planar projection boundary of the actual hazardous area. Second, the coal yard height parameter is superimposed to form a vertical constraint, creating a configurable 3D clipping box. This area division method adapts to the horizontal characteristics of the coal yard ground, avoids redundant area extraction algorithms, and supports the dynamic adjustment of the base-plane vertex coordinates, which can change with the work area. This method can effectively enhance the flexibility of establishing intrusion judgment methods. Based on the above means, the 3D information of the hazardous area can be obtained.

**Figure 6:**
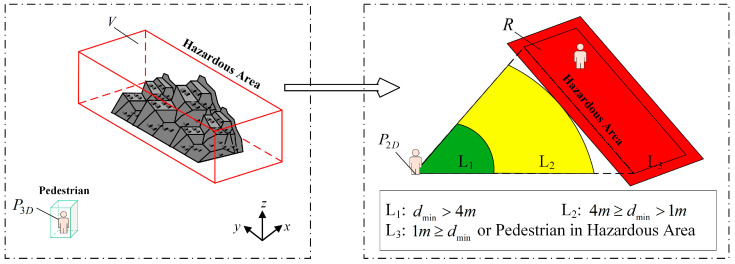
**Three-dimensional** space pedestrian intrusion judgment. When the distance between a pedestrian and the hazardous area is $d_{\min}>4m$, the pedestrian is in the green $L_{1}$ level; when the distance is $4m\geq d_{\min}>1m$, the pedestrian is in the yellow $L_{2}$ level; when $1m\geq d_{\min}$ or the pedestrian is within the hazardous area, the pedestrian is in the red $L_{3}$ level.

In an actual coal yard, the ground can be approximately regarded as an ideal horizontal plane. This assumption provides theoretical feasibility for the dimensionality reduction of 3D spatial relationships. Based on this, this study uses the orthogonal projection method to map the object pedestrian and hazardous areas in the same coordinate system onto a 2D plane, as shown in Figure 6.

The specific description is as follows: Let the coordinates of the center point of the 3D detection box of the object pedestrian be $P_{3D}=(x_{p},y_{p},z_{p})$, the size be $S_{3D}=(w,l,h)$, and the set of bottom vertices of the 3D polygon of the hazardous area be $\left\{V_{r}=(x_{r},y_{r},z_{r})\left|r=1,2,3,4\right.\right\}$(the base-plane polygon constituting the dangerous area can be arbitrary; here, we choose to use a quadrilateral derivation). Since the ground is horizontal, by establishing the projection operator $\prod :{\mathbb{R}}^{3}\to {\mathbb{R}}^{2}$, we can obtain the following:

(8) $$\prod (P_{3D})=(x_{p},y_{p}),\;\;\prod (V_{r})=(x_{r},y_{r}),\;\;r=1,2,3,4$$

After projection, the coordinates of the object pedestrian points are $P_{2D}=(x_{p},y_{p})$, and the hazardous area forms a polygon R. This mapping process preserves the topological relationships in 3D space and simplifies the intrusion problem in the analysis of the point–polygon positional relationship in a 2D plane, significantly reducing the complexity of the algorithm. Based on this, the research proposes a pedestrian intrusion early warning model, which constructs $L_{1}$, $L_{2}$, and $L_{3}$ hierarchical early warning strategies through a hierarchical judgment method. First, the ray casting algorithm [42] is used to determine whether a point is inside or outside; then, the minimum distance from an external point to the polygon R is calculated.

**Determination of the positional relationship between a point and a polygon:** A closed polygon in the plane divides it into interior and exterior regions. When a straight line intersects the polygon’s boundary, it either enters or moves away from the polygon. Therefore, this study uses the ray casting algorithm to determine whether a personnel target exists in the hazardous area. The occurrence of intrusion in the polygonal area is determined based on the following equation:

(9) $$\chi =sum[q(P_{2D},n)\%2]$$

Here, q represents the emission of n rays from the point $P_{2D}$ in arbitrary directions, %2 denotes the remainder when divided by 2, sum represents the summation, and χ indicates whether a regional intrusion has occurred. When the number of intersections of the rays is odd, the target person is within the dangerous area; otherwise, the target person is outside the dangerous area. In special cases, when the point falls on the boundary of the polygon, it is still judged as $L_{3}$, indicating that an intrusion has occurred.

**Calculation of the shortest distance to external points:** When the object pedestrian is outside the hazardous area, the problem is transformed into finding the shortest distance d from a point to each side of the polygon, which is also equivalent to calculating the length of the line segment equation V(t). Finally, by comparing the lengths of each line segment equation, the shortest distance $d_{\min}$ from the external point $P_{2D}$ to the rectangle R can be obtained. V(t) is expressed as follows:

(10) $$V(t)=V(P_{2D},t\cdot \vec{AB})$$

(11) $$t=\frac{\vec{AB}\cdot \vec{AP_{2D}}}{{\left\|AB\right\|}^{2}}$$

Here, V(t) represents the distance line segments from an external point to each side of the polygon, $\vec{AB}$ is the vector of any side of the rectangle, $A=(x_{1},y_{1})$, $B=(x_{2},y_{2})$, and t is the projection parameter, which can be determined using the line segment inner product formula. There are three cases for V(t), which can be solved by calculating the projection distance from a point to a line, since the length of V(t) is the same as the value of d, as shown in Equation (12).

(12) $$d=\left\{\begin{array}{c} \sqrt{{\left(x_{p}-x_{1}\right)}^{2}+{\left(y_{p}-y_{1}\right)}^{2}}\\ \sqrt{{\left(x_{p}-x_{c}\right)}^{2}+{\left(y_{p}-y_{c}\right)}^{2}}\\ \sqrt{{\left(x_{p}-x_{2}\right)}^{2}+{\left(y_{p}-y_{2}\right)}^{2}} \end{array}\right.$$

The shortest distance $d_{min}$ from point $P_{2D}$ to polygon R can be obtained using the above method; this is also the shortest distance from the object pedestrian to the hazardous area. Using the distance relationship between the two, a hierarchical progressive intrusion judgment model is established: $d_{min}>4m$ serves as the prejudge layer ($L_{1}$); $4m\geq d_{min}\geq 1m$ serves as the warning layer ($L_{2}$); $1m\geq d_{min}$, and the result indicating that the pedestrian is within the hazardous area serves as the alarm layer ($L_{3}$). While it is simpler to implement a single expanded warning zone, this inevitably leads to high false alarm rates and alarm fatigue because workers often operate near hazardous areas. Our tiered approach mitigates this problem by providing graded warnings based on precise hazard distance metrics.

## 3. Experiments and Results

### 3.1. Pedestrian Object Experiments Using a Public Dataset

#### 3.1.1. Dataset

**Figure 7:**
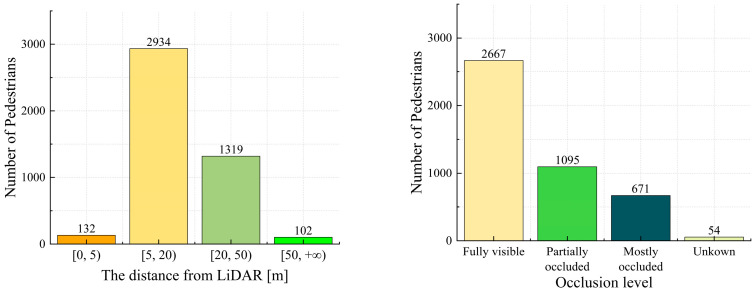
Geometric statistics of pedestrian instances in the KITTI dataset under different distance and occlusion distributions. The pedestrian category in the KITTI dataset encompasses various states of pedestrian targets, which helps to enhance the generalization ability and robustness of the pedestrian target detection model.

The KITTI dataset [43] is commonly used in research on 3D object detection. This dataset includes data from various target objects collected using color cameras, grayscale cameras, and LiDAR, recorded in a real traffic environment over a six-hour period. The dataset consists of 7481 training and 7518 testing point cloud data points. The KITTI 3D object dataset is primarily utilized for 3D object detection, containing numerous 3D point cloud, annotation, and calibration files from real-world scenarios. The detection categories include Car, Pedestrian, Truck, Van, and Cyclist. There are approximately 4500 training instances of Pedestrians with 3D labels. This study mainly evaluated the Pedestrian category. The geometric statistics of the distance distribution and occlusion distribution of the pedestrian category in the dataset were obtained by processing all the label files (label_2/*.txt) in the KITTI dataset, as shown in Figure 7. The pedestrian category contains instances with different distances and occlusion conditions; therefore, it can accurately represent pedestrian targets in real working conditions. This study used this data as the training sample for object detection.

#### 3.1.2. Experimental Setup and Evaluation Metric

The experimental hardware configuration comprises a 64-bit Linux system (Ubuntu 22.04), an NVIDIA H800 GPU, and an Intel(R) Xeon(R) Gold 6248R CPU. The experimental environment consists of Python 3.9, CUDA 11.8, Torch 2.1, and spconv 2.3.6. The experiment adopts a point cloud range of [0, 70.4] m along the *x*-axis, [−40, 40] m along the *y*-axis, and [−3, 1] m along the *z*-axis, with an input point cloud voxel size of [0.05, 0.05, 0.1] m. The EFT-RCNN model is trained on a single GPU. The model is trained for 80 epochs with a batch size of 8. The Adam-onecycle optimizer is used for network optimization, with an initial learning rate of 0.01 and an optimization momentum parameter of 0.9. The anchor box for the pedestrian category is set to [0.8, 0.6, 1.73] m.

In this study, the model is evaluated from two main perspectives—accuracy and operational efficiency—following the latest KITTI standard method. We focus solely on the pedestrian category, which is consistent with the application scenario. The average precision (AP, %) metric is used in this study to evaluate the performance of the object detection model for pedestrian detection, regardless of other categories. Different Intersection over Union (IoU) values, set at 0.5 and 0.25, were used as matching criteria. The experimental settings followed the evaluation rules of the KITTI dataset benchmark test; that is, when the IoU thresholds were set at 0.5 and 0.25, the AP was used to evaluate the performance of the pedestrian detector at three levels: easy, moderate, and hard. This experimental setup provides a comprehensive assessment of the detector’s detection accuracy under different conditions, which is crucial for safety monitoring systems. The study uses Frames Per Second (FPS) to evaluate the operational efficiency of the model.

**IoU:** This refers to the ratio of the intersection to the union of the predicted bounding box and the ground truth bounding box, which characterizes the degree of overlap between the two. A larger value indicates more overlap between the two boxes and a more accurate detection result. The calculation formula is as follows:

(13) $$IoU=\frac{\left|V_{p}\cap V_{g}\right|}{\left|V_{p}\cup V_{g}\right|}$$

Here, $V_{p}$ represents the volume of the predicted 3D bounding box, and $V_{g}$ is the volume of the ground truth 3D bounding box. This formula is used to calculate the IoU between the 3D predicted bounding box and the ground truth bounding box. The calculation of the IoU for the BEV and 2D bounding boxes is analogous to the above formula.

**AP:** KITTI divides the real categories and detection categories of data annotation into True Positive (TP), False Positive (FP), True Negative (TN), and False Negative (FN), which represent the correctly detected positive class, the correctly detected negative class, the incorrectly detected positive class, and the incorrectly detected negative class, respectively.

(14) $$P=\frac{TP}{TP+FP}$$

(15) $$R=\frac{TP}{TP+FN}$$

Here, precision (P) is the proportion of true positives among the correctly predicted cases, and recall (R) is the proportion of true positive cases among all positive samples.

In object detection, IoU is often used to determine TP, FP, FN, and TN, and then calculate P and R. In this study, different IoUs (0.5, 0.25) are selected. When calculating AP, all prediction results are first arranged in descending order of confidence. Then, the values of *P* and *R* are calculated according to different confidence thresholds to obtain the *P*-*R* curve. The value of AP is the area under the *P*-*R* curve for a single category. The interpolation method is usually used to calculate this area to avoid oscillations in the *P*-*R* curve. The calculation formula is as follows:

(16) $$\rho_{i}(r)=\underset{\tilde{r}:\tilde{r}\geq r}{\max}$$

(17) $$AP\left|R_{N}=\frac{1}{N}\right.\sum_{r\in R_{N}}\rho_{i}(r)$$

Here, $\rho_{i}(r)$ is the interpolation function. In this study, the latest calculation standard for the KITTI dataset is adopted, and the R coordinates are divided into 40 equidistant recall levels. $AP\left|R_{N}\right.$ gives a more accurate AP by calculating the average value of P at these 40 recall rates.

**FPS:** The formula for calculating FPS is as follows:

(18) $$FPS=\frac{1}{T_{pre}+T_{inf}+T_{post}}$$

Here, $T_{pre}$ represents the pre-processing time for a single-frame point cloud, $T_{inf}$ denotes the inference time, and $T_{post}$ stands for the post-processing time.

#### 3.1.3. Experimental Results of the Application of the EFT-RCNN Network to a Public Dataset

To comprehensively evaluate the performance of the detection algorithm EFT-RCNN, the official KITTI benchmark dataset was used for evaluation. For a comprehensive comparison, several widely recognized and state-of-the-art object detection algorithms were selected for the experiments, and all experimental parameters were set consistently. As shown in Table 1, compared with existing methods, EFT-RCNN achieves a 4.39% increase in 3D AP compared with the baseline model Voxel-RCNN, and outperforms all the comparison methods, indicating its stronger comprehensive detection ability under different IoU difficulty thresholds. Under the standard condition of an IoU of 0.5, EFT-RCNN achieved the highest AP values across the three pedestrian detection difficulty levels: easy, moderate, and hard. EFT-RCNN achieved the second-highest AP value under the loose condition of an IoU of 0.25. Sub-optimal AP values were obtained at the three difficulty levels; thus, the model proposed has higher detection accuracy under standard conditions compared with other detectors, and has a better recall rate under loose conditions. EFT-RCNN outperforms the latest PDV and HINTED models in terms of FPS, showing an improvement of nearly two times. Additionally, compared with the classical Part-A2 and PV-RCNN detection models, EFT-RCNN improves the 3D AP by 2.26% and 3.43%, respectively, while maintaining a similar inference speed, reflecting the advantage of the algorithm in terms of computational efficiency.

Ablation experiments were designed to verify the effects of EnhancedVFE, 3D FocalConv, and TeBEVPooling in improving detection accuracy and operational efficiency. The baseline model used in the study is Voxel-RCNN, and five groups of experiments were designed. Each group of experiments used the same experimental settings. The impacts of different improvement methods on object detection are shown in Table 2.

For experiment (a), the 3D AP and BEV AP are improved by 2.67% and 3.35%, respectively, compared with the baseline. EnhancedVFE can capture rich geometric information in the 3D space and enhance the voxel feature extraction ability, significantly improving the positioning accuracy of the detection boxes. For experiment (b), the 3D AP and BEV AP are improved by 1.99% and 2.16%, respectively, compared with the baseline. Three-dimensional FocalConv enhances the expression of important features and suppresses the interference of unimportant features through FocalConv, effectively reducing the interference of the background on the foreground pedestrian objects and improving the model detection accuracy. For experiment (c), the 3D AP and BEV AP are improved by 2.68% and 2.85%, respectively, compared with the baseline. TeBEVPooling aggregates multi-transformed features through max-pooling, effectively highlighting the key features of pedestrians and suppressing the complex background noise. Moreover, this method directly optimizes the generation of the BEV, effectively improving the BEV AP. However, while improving the detection accuracy, all three improvements have a negative impact on the computational efficiency, especially Improvement 2, which is caused by the complex network structure of FocalConv. By comprehensively applying these three improvements, the EFT-RCNN model proposed in this study achieves improvements of 4.39% and 4.68% in 3D AP and BEV AP, respectively, compared with the baseline. The improvement in 3D AP benefits from the enhanced feature representation of EnhancedVFE and the foreground focusing ability of 3D FocalConv, which together optimize object localization in 3D space. The improvement in BEV AP is attributed to the noise suppression and geometry preservation characteristics of TeBEVPooling. Combined with the rich feature input of EnhancedVFE, the robustness of the detection boxes under BEV is significantly improved.

**Figure 8:**
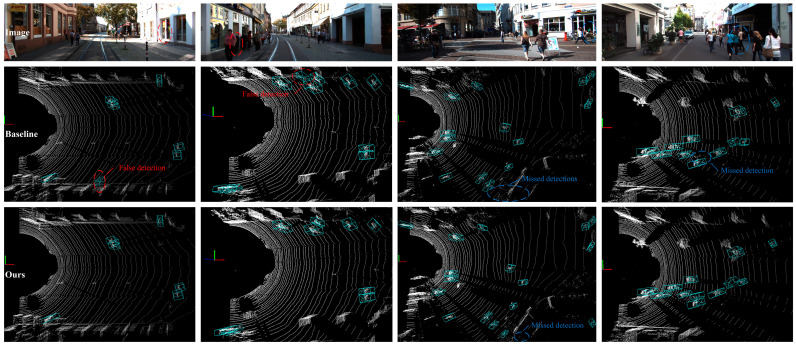
Comparison of pedestrian detection by the baseline model and the EFT-RCNN using the KITTI val set. Four scenarios from the KITTI val set were chosen for analysis. The initial row exhibits the actual images for these scenarios, the subsequent row shows the baseline model’s detection outcomes, and the final row shows the detection results of the EFT-RCNN proposed in this study. False detections are circled in red, and missed detections are circled in blue.

To more intuitively evaluate the detection performance, Figure 8 presents the test results of two models. In Scenarios 1 and 2, the baseline model made false detections: in Scenario 1, the baseline model misidentified the ghost image of a pedestrian reflected inside a glass door as a real pedestrian, primarily due to reflection and light transmission, while in Scenario 2, the baseline model misidentified a flower bed as a pedestrian. In contrast, the EFT-RCNN effectively identified all pedestrians in the two scenarios. In Scenario 3, where the background was most complex, the baseline model significantly failed to detect pedestrians, and the EFT-RCNN also failed to detect a pedestrian who was far away and indoors. In Scenario 4, characterized by overlapping and occluded pedestrians, the baseline model exhibited missed detections, whereas the EFT-RCNN successfully detected all pedestrians. Therefore, the EFT-RCNN significantly improves the performance of the pedestrian detection network by reducing false detections and missed detections in complex backgrounds and slight occlusion situations.

In pedestrian intrusion detection in hazardous areas, missed detection and false detection may lead to serious safety-related accidents. Compared with other detectors, EFT-RCNN has higher detection accuracy under difficult sample conditions, which can effectively reduce the risk of missed detection and false detection in complex environments. It also exhibits high recall ability under loose IoU conditions, thereby reducing the missed detections caused by sparse and occluded pedestrian targets. In addition, its balanced real-time performance meets the basic requirements of the actual detection system for response speed, and has high engineering realization potential.

### 3.2. On-Site Pedestrian Intrusion Detection Experiments

#### 3.2.1. On-Site Deployment

**Figure 9:**
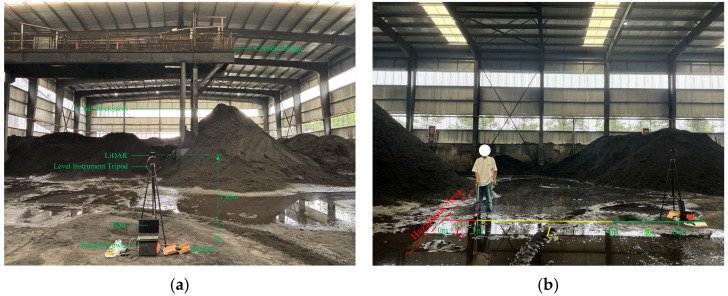
Installation of hardware equipment: (**a**) the test equipment consists of a LiDAR (QT128), a level instrument tripod, an adapter, a computer host, a portable power supply, and various cables; (**b**) the relationship between the LiDAR and the location of the hazardous area.

To verify the effectiveness of the proposed pedestrian object detection and point–region hierarchical judgment method in the actual coal yard environment, a LiDAR sensor (QT128 [47]; the sensor parameters are shown in Table 3) was installed in a coal yard to collect real-time 3D point cloud environmental data. The on-site dataset collection lasted two days, yielding a total of 15 point cloud sequences (approximately 6 GB). These sequences covered varied conditions, including different pedestrian densities, occlusion levels, and lighting conditions, representing typical operational scenarios in the coal yard. This dataset was used to test the detection performance of the method and verify the false detections and missed detections of the method in real-world scenarios. QT128 was connected to one end of the adapter, and the other end of the adapter was connected to the host and the portable power via an Ethernet cable and a power cord, respectively, as shown in Figure 9. The network parameters of the host were configured to ensure that its IP address is in the same subnet as that of QT128, thereby facilitating real-time point cloud data transmission. The experimental site was the coal yard. Raw coal was transported to the ground via a conveyor belt installed on a corridor bridge to form coal piles. Depending on site needs, areas below and around the coal piles are defined as high-risk areas where workers are exposed to significant safety risks. The LiDAR was set as the origin of the coordinate system and was installed at a height of 1.85 m above the ground. The direction from the LiDAR to the corridor bridge was defined as the *x*-axis, and the left-hand side was defined as the *y*-axis. The distance from the LiDAR to the boundary of the hazardous area was measured, and the coordinates of each vertex of the base polygon were determined through exploration as follows: (18.0, 11.0), (18.0, −6.0), (12.0, −12.0), (6.0, −12.0), (6.0, 11.0), with a constrained height of 3 m. The warning thresholds (L1, L2, L3) and hazardous area polygon are fully configurable, allowing adaptation to different yard layouts and safety requirements. Similarly, the installation height and position of LiDAR only affect the coordinate system and the definition of the dangerous area, which are configurable.

**Figure 10:**
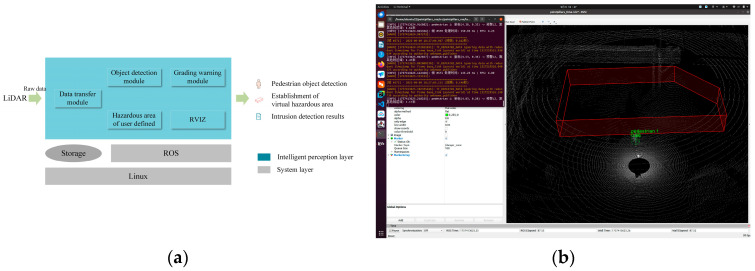
Algorithm deployment and testing. (**a**) Design of ROS-based system software architecture; (**b**) RVIZ visualization test results.

**Figure 11:**
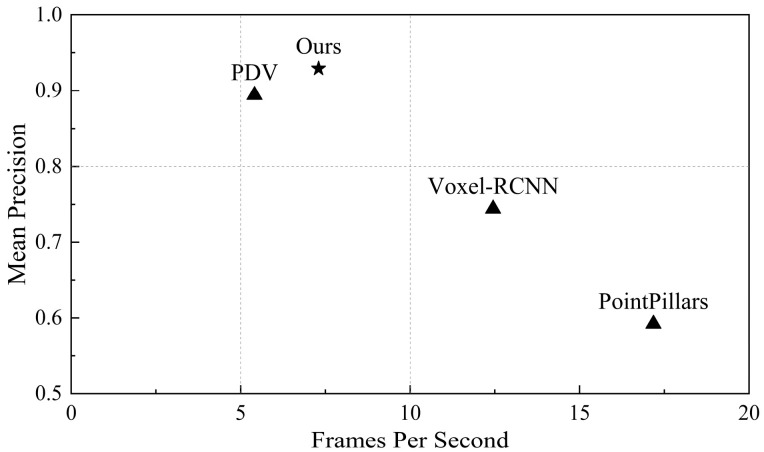
Performance comparisons. This plot shows the mean Precision of the model versus FPS in on-site testing. We compared our model with existing 3D object detection models, which were tested on hardware platforms such as the 13th Gen Intel Core i9-13900HX (2.20 GHz) and NVIDIA GeForce RTX 4060 (8 GB).

As shown in Figure 10a, the algorithm in this study is deployed and implemented based on ROS. The experimental deployment environment consists of Ubuntu 20.04, ros-noetic, cuda11.8, Python3.9, and Torch2.1. ROS is used to subscribe to the real-time point cloud messages of QT128. The pretrained EFT-RCNN model is run for pedestrian detection, and the detection boxes and category labels are output. The hazardous areas are divided based on the preset polygonal prisms. The hierarchical early-warning algorithm is executed to calculate the distance relationship between the pedestrian objects and the hazardous area and determine the early-warning level. Finally, ROS is used to publish the detection boxes, labels, virtual hazardous area bounding boxes, and early-warning information. As shown in Figure 10b, the ROS tool RVIZ is utilized to visualize the original point cloud, pedestrian detection boxes, labels, and the 3D bounding boxes of the virtual hazardous area in real time. Meanwhile, the system terminal synchronously outputs detailed data for each frame, including the detection status, the 3D coordinates of pedestrian objects, the minimum distance to the hazardous areas, the triggered early-warning level, and the processing time per frame. The on-site test is shown in Figure 11. We implemented the deployment on a laptop equipped with a 13th Gen Intel Core i9-13900HX processor (2.20 GHz), an NVIDIA GeForce RTX 4060 (8 GB), and 32 GB of RAM. The baseline algorithm, Voxel-RCNN, was also tested in the same environment.

#### 3.2.2. Results of Pedestrian Intrusion Detection in the Coal Yard Environment

To comprehensively evaluate the performance of the EFT-RCNN model in a real coal yard environment, this study conducted on-site deployment tests in the coal pile loading area under the coal yard gallery bridge. We randomly recorded three representative point cloud sequence data—Scenario A, B, and C—to present the actual intrusion scenarios. All scenes collected in the coal yard include lighting and occlusion under different conditions. Scenario A (Single-person scenario): One pedestrian moves from the periphery of the hazardous area to the inside to test the model’s ability to detect a single object. Scenario B (Two-person scenario): Two pedestrians enter the monitoring area simultaneously, with partial occlusion and interaction behaviors, to test the detection robustness of the model under multi-target and mild occlusion conditions. Scenario C (Multi-person scenario): Three people are active near and inside the hazardous area, where the objects are dense and severely occluded by each other, to test the performance limit of the model in a complex interaction environment.

For these three scenarios, we evaluate the proposed EFT-RCNN and various advanced models (including baseline network Voxel-RCNN, PointPillars, and PDV) from both Precision and FPS aspects, as shown in Table 4. In terms of algorithm accuracy, EFT-RCNN achieved the highest detection accuracy of 92.9% among all contrast models. In terms of algorithm efficiency, PointPillars achieved the highest FPS; the detection efficiency of EFT-RCNN was 7.3 FPS. Figure 11 shows the results of this comparison. FET-RCNN strikes a balance between accuracy and acceptable real-time performance, making it suitable for safety-oriented applications in areas such as safety monitoring. For some applications that require high efficiency and can sacrifice accuracy, methods such as PointPillars and Voxel-RCNN can be considered. This efficiency difference stems primarily from the fact that the actual deployed system needs to receive the original point cloud data stream from the LiDAR in real-time through the CPU, and execute the complete early-warning workflow simultaneously. Despite these additional system processing environments, the algorithm in this study meets the requirements of real-time monitoring applications for pedestrian movement scenarios under actual deployment.

**Figure 12:**
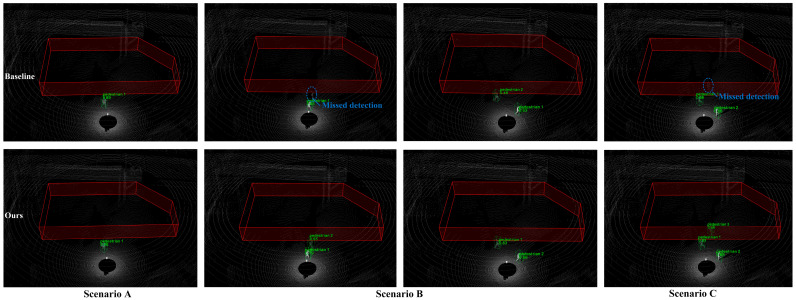
Comparison of actual detection effects of different models in real scenarios. Missed detections are circled in blue. The red polygon prism is the hazardous area, the green box is the pedestrian results, and the text above is the category label. The baseline model fails to detect pedestrians in scenarios with severe occlusion in Scenario B and sparse human point clouds in Scenario C. In contrast, the model proposed in this study achieves accurate detection in all cases.

We compare the baseline network and EFT-RCNN detection results under three scenarios, as shown in Figure 12. The Voxel-RCNN model exhibits obvious missed detection problems in complex scene environments. The missed detections are mainly caused by pedestrian overlap, which is particularly severe in scenarios with occlusions (Scenario B) or multiple people (Scenario C). The proposed network enhances geometric feature extraction in 3D space through EnhancedVFE, improves detection ability under sparse point clouds, and effectively suppresses complex background through FocalConv-focused foreground human target extraction. These improvements enhance the robustness of detection. In the figure, EFT-RCNN can detect all pedestrians in the scene without interference from sparse point clouds, occlusion, background environments, and other factors.

#### 3.2.3. Static Pedestrians Grading Judgment Results in the Coal Yard

**Figure 13:**
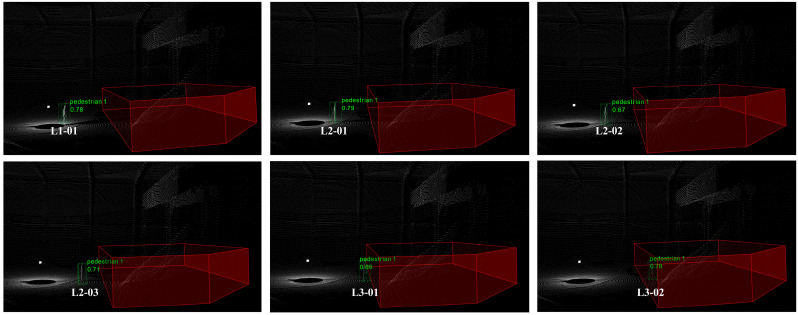
Multi-location static pedestrian detection results. The red polygon prism is the hazardous area, the green box is the pedestrian results, and the text above is the category label.

A static pedestrian point cloud test experiment was designed to quantitatively evaluate the reliability of warning level determination and the spatial positioning accuracy of the point–region hierarchical judgment method. As shown in Figure 13, 2, 3, and 2 pedestrians to be detected were randomly placed in fixed locations within the three preset levels L1, L2, and L3. A total of 7 locations and 7 segments of point cloud sequence data were recorded. A measuring instrument was used to measure the distance between the pedestrian at each point and the boundary of the hazardous area as the ground truth. The warning level and the measured distance value of each point were output in real-time through the algorithm. The core data are summarized in Table 5.

The results indicate that, in terms of warning hierarchical judgment, the algorithm accurately identifies the warning levels of all locations. In terms of distance measurement accuracy, the measurement errors of all external points do not exceed 0.1 m, and the average error is only 0.054 m. This high-precision distance information provides a reliable basis for implementing hierarchical warning strategies such as “proximity warning” and “intrusion warning”, significantly improving the accuracy and timeliness of warnings.

## 4. Discussion and Conclusions

The intrusion of workers into the coal stockyard area is the main cause of safety-related accidents in coal yards. Traditional visual detection methods are significantly affected by environmental interference, making it difficult to accurately express the positional relationship between the pedestrian and the hazardous area, and the detection effect is passive. This study proposed a method for detecting pedestrian intrusion into hazardous areas based on LiDAR point clouds, which enables “active prevention” of intrusion into hazardous areas, effectively compensates for the shortcomings of traditional detection methods, accurately identifies pedestrian objects, and expresses the pedestrian’s positional relationship with the hazardous area.

The main contributions of this study are as follows: (1) A robust 3D pedestrian object detection network, EFT-RCNN, was proposed. Through targeted improvements in the baseline network structure, it effectively overcomes the interference of the coal yard’s complex background. Using the public dataset, the 3D AP of the detection reached 64.93%. Additionally, it maintained a frame rate of 28.56 FPS, achieving a balance between detection accuracy and efficiency. In the on-site coal yard environment, the average detection accuracy under different conditions was 92.9%, providing key technical support for reliable pedestrian perception in complex industrial scenarios. (2) A point–region hierarchical judgment method was proposed. This method calculates the intrusion distance based on accurate 3D position information, overcoming the limitations of traditional single-step judgment. The average error in the measured distance was only 0.054 m, realizing quantitative assessment and early warning of intrusion threats and significantly enhancing safety. (3) The system deployment and verification were completed based on ROS, providing a reference for engineering of the 3D point cloud object detection algorithm in actual industrial scenarios. It also provides a new method for regional intrusion detection.

However, this study has several limitations, providing guidance for future improvements. First, for pedestrian bodies with abnormal movements or severe occlusions, the accuracy of the object detection algorithm needs to be improved. Using a targeted dataset to replace the public dataset for training is a potential way of addressing this issue. Second, there is significant room to improve the processing speed of the algorithm in actual deployment. Downsampling the input point cloud data stream and using tools such as TensorRT to accelerate model inference can be adopted. Finally, the algorithm can be deployed on the NVIDIA Jetson series to evaluate its real-time performance under resource-constrained conditions and better describe its scaling behavior.

## Figures and Tables

**Table 1:** Comparison of the experimental results of different models. Pedestrians@0.5 AP represents the AP values of 3D detection results under easy, moderate, and difficult conditions when the IoU is 0.5; 3D AP represents the average value of the AP of 3D detection boxes under different IoUs. Bold font represents the optimal indicators, and underlined font represents the sub-optimal indicators.

Method	3D AP	Pedestrians@0.5 AP			Pedestrians@0.25 AP			FPS
		Easy	Moderate	Hard	Easy	Moderate	Hard	
SECOND [22]	57.87	51.84	45.57	40.81	74.24	70.08	66.65	36.10
PointPillars [23]	55.58	49.62	43.51	38.50	71.40	67.15	63.28	**58.14**
PillarNet [24]	57.29	47.29	41.57	37.69	77.07	71.80	68.31	55.56
PV-RCNN [26]	61.50	56.56	49.35	44.82	77.58	71.77	68.94	29.24
Part-A2 [27]	62.67	60.81	52.22	46.67	77.48	71.47	67.36	35.46
Voxel-RCNN [29]	60.54	58.91	51.95	47.15	72.78	68.09	64.37	55.87
OcTr [44]	62.92	59.67	52.43	46.83	78.29	71.76	68.54	32.62
PDV [45]	64.59	60.92	54.06	48.25	**79.74**	**74.16**	**70.40**	16.18
HINTED [46]	62.10	60.32	53.51	47.38	76.74	70.49	64.14	18.05
Ours	**64.93**	**63.50**	**54.31**	**49.43**	79.51	72.97	69.83	28.56

**Table 2:** Comparison results of ablation experiments. The meanings of 3D AP and FPS are the same as in Table 1, and BEV AP represents the average value of AP for BEV detection boxes under different IoUs. Improvements 1, 2, and 3 represent EnhancedVFE, 3D FocalConv, and TeBEVPooling, respectively. ✓ indicates that the baseline model has undergone this improvement.

Method	Improvement 1	Improvement 2	Improvement 3	3D AP	BEV AP	FPS
Voxel-RCNN				60.54	62.19	55.87
(a)	✓			63.21	65.54	34.48
(b)		✓		62.53	64.35	30.58
(c)			✓	63.22	65.04	44.84
Ours	✓	✓	✓	64.93	66.87	28.56

**Table 3:** QT128 specifications.

Parameter	Value
range capability	20 m @10% reflectivity
point rate	864,000 (single return)
field of view	360° * 105°
angular resolution	0.4°(H) * 0.4°(V)
range accuracy	±2 cm

**Table 4:** Comparison of detection results of different models in real scenarios. Under scenarios A, B, and C, the performance of four detection models in terms of accuracy and efficiency was compared.

Metric	Detection Method	Scenario A	Scenario B	Scenario C	Average Value
Precision (%)	Baseline	79.6	72.9	70.8	74.4
	PointPillars	63.7	58.6	55.2	59.2
	PDV	92.4	89.3	86.5	89.4
	Ours	97.0	92.1	89.6	**92.9**
FPS	Baseline	12.56	11.28	13.51	12.45
	PointPillars	18.32	16.40	16.83	**17.18**
	PDV	5.64	5.31	5.28	5.41
	Ours	7.42	7.13	7.36	7.30

**Table 5:** Measurement results of pedestrian hierarchical judgment. * L3-02 is within the dangerous area, so there is no error calculation result.

Location ID	Warning Level	Ground Truth (m)	Predicted Value (m)	Error (m)
L1-01	L1	4.50	4.45	0.05
L2-01	L2	3.50	3.54	0.04
L2-02	L2	3.00	3.07	0.07
L2-03	L2	2.00	2.03	0.03
L3-01	L3	0.50	0.62	0.08
L3-02 *	In the hazardous area	-	-	-

## Data Availability

The KITTI dataset presented in the study is openly available at https://www.cvlibs.net/datasets/kitti/ (accessed on 12 August 2025). The original contributions presented in this study are included in the article. Further inquiries can be directed to the corresponding authors.
